# Delayed Rectifier K^+^-Channel Is a Novel Therapeutic Target for Interstitial Renal Fibrosis in Rats with Unilateral Ureteral Obstruction

**DOI:** 10.1155/2019/7567638

**Published:** 2019-11-07

**Authors:** Nozomu Abe, Hiroaki Toyama, Kazutomo Saito, Yutaka Ejima, Masanori Yamauchi, Hajime Mushiake, Itsuro Kazama

**Affiliations:** ^1^Department of Anesthesiology, Tohoku University Hospital, Seiryo-cho, Aoba-ku, Sendai, Miyagi, Japan; ^2^Department of Physiology, Tohoku University Graduate School of Medicine, Seiryo-cho, Aoba-ku, Sendai, Miyagi, Japan; ^3^Miyagi University, School of Nursing, Gakuen, Taiwa-cho, Kurokawa-gun, Miyagi, Japan

## Abstract

**Background:**

Delayed rectifier K^+^-channel, Kv1.3, is most predominantly expressed in T-lymphocytes and macrophages. In such leukocytes, Kv1.3-channels play pivotal roles in the activation and proliferation of cells, promoting cellular immunity. Since leukocyte-derived cytokines stimulate fibroblasts to produce collagen fibers in inflamed kidneys, Kv1.3-channels expressed in leukocytes would contribute to the progression of tubulointerstitial renal fibrosis.

**Methods:**

Male Sprague-Dawley rats that underwent unilateral ureteral obstruction (UUO) were used at 1, 2, or 3 weeks after the operation. We examined the histological features of the kidneys and the leukocyte expression of Kv1.3-channels. We also examined the therapeutic effects of a selective channel inhibitor, margatoxin, on the progression of renal fibrosis and the proliferation of leukocytes within the cortical interstitium.

**Results:**

In rat kidneys with UUO, progression of renal fibrosis and the infiltration of leukocytes became most prominent at 3 weeks after the operation, when Kv1.3-channels were overexpressed in proliferating leukocytes. In the cortical interstitium of margatoxin-treated UUO rat kidneys, immunohistochemistry revealed reduced expression of fibrosis markers. Additionally, margatoxin significantly decreased the numbers of leukocytes and suppressed their proliferation.

**Conclusions:**

This study clearly demonstrated that the numbers of T-lymphocytes and macrophages were markedly increased in UUO rat kidneys with longer postobstructive days. The overexpression of Kv1.3-channels in leukocytes was thought to be responsible for the proliferation of these cells and the progression of renal fibrosis. This study strongly suggested the therapeutic usefulness of targeting lymphocyte Kv1.3-channels in the treatment of renal fibrosis.

## 1. Introduction

Chronic tubulointerstitial nephritis (TIN) is an entity of renal disease characterized by a progressive scarring of tubulointerstitium [1], sometimes deteriorating into end-stage renal disease [2, 3]. The lesion includes tubular atrophy, leukocyte infiltration, and interstitial fibrosis. In addition to drugs and toxins, such as analgesics, antibiotics, Chinese herbs, and heavy metals [4–6], chronic ureteral obstruction and repetitive infection are also the leading causes of chronic TIN, especially in infants [7, 8]. To reproduce the lesion characteristic to renal fibrosis, the animal model of unilateral ureteral obstruction (UUO) was developed in 1970s, which primarily represented the pathology of obstructive nephropathy [9]. In rodent models of UUO, leukocytes, such as lymphocytes, macrophages, neutrophils, and mast cells, are known to infiltrate into the renal interstitium [9–11]. Among them, many studies have focused on the involvement of mast cells in the development of renal fibrosis [12–14], in which mast cells were demonstrated to produce fibroblast-activating factors in addition to chemical mediators [15, 16]. However, we know little about the pathological roles of T-lymphocytes or macrophages in the progression of renal fibrosis, despite their predominance in the renal interstitium [9–11]. These leukocytes principally express delayed rectifier K^+^-channels (Kv1.3) in their plasma membranes, and the channels play critical roles in the activation and proliferation of the cells [17, 18]. Since the cytokines produced by the inflammatory leukocytes directly stimulate the collagen synthesis from interstitial fibroblasts [19], the channels expressed in the leukocytes would contribute to the progression of renal fibrosis in UUO. To clarify this, using a rat model of UUO, we examined the Kv1.3-channel expression in the kidneys and the therapeutic effects of a selective channel inhibitor, margatoxin, on the progression of renal fibrosis and the proliferation/activation of leukocytes there. Here, we clearly show that the numbers of T-lymphocytes and macrophages were markedly increased in UUO rat kidneys at 3 weeks after the operation. We also show that the overexpression of Kv1.3-channels in leukocytes was responsible for the proliferation of these cells and the progression of renal fibrosis. This study strongly suggests the therapeutic usefulness of targeting lymphocyte Kv1.3-channels in the treatment of renal fibrosis.

## 2. Materials and Methods

### 2.1. Animal Preparation and UUO Induction

Male Sprague-Dawley rats weighing 150–180 g (Japan SLC Inc., Shizuoka, Japan) underwent unilateral ureteral ligation, as described in previous studies [9–11]. Briefly, after the rats were deeply anesthetized with isoflurane, the left ureter was exposed through a lateral flank incision. Then the ureter was ligated with 3-0 silk at two points under sterile conditions. During the subsequent 1 to 3 weeks, rats had free access to standard rat chow and water ad libitum and were maintained in a humidity- and temperature-controlled room on a 12-hour light-dark cycle. One, two, or three weeks after the operation, the rats were deeply anesthetized and then killed by cervical dislocation. The left kidneys were removed for histological examination and RNA extraction. The contralateral kidneys at 3 weeks after the operation were used as controls. Trunk blood was withdrawn for the measurements of serum creatinine and urea nitrogen levels. All experimental protocols described in the present study were approved by the Ethics Review Committee for Animal Experimentation of Tohoku University.

### 2.2. Margatoxin Treatment

For the treatment with Kv1.3-channel inhibitor, margatoxin (Peptide Institute, Osaka, Japan) was dissolved in normal saline to prepare a concentration of 200 nM. After inducing unilateral ureteral obstruction, the rats were intraperitoneally injected with 200 nM/ml margatoxin daily for 3 weeks (margatoxin-treated group). In our previous study, using rat models with advanced chronic renal failure (CRF), 100 nM/ml margatoxin actually ameliorated the progression of renal fibrosis without causing any adverse events [20]. In our preliminary study, since 100 nM margatoxin did not ameliorate the progression of renal fibrosis in UUO rat kidneys, we selected higher dose in the present study. At the end of the observation period, the left kidneys were removed for histological examination and RNA extraction.

### 2.3. Histological Analyses

Renal cross sections were fixed in 4% paraformaldehyde, embedded in paraffin, and deparaffinized in xylene, and then 3 *μ*m sections were stained with hematoxylin-eosin (H&E) and Masson's trichrome. For fibrosis analysis, Masson's trichrome deposition, expressed as percentages of Masson's trichrome-positive areas relative to the total area, was quantified in each field and averaged, as described in our previous studies [16, 21–23].

### 2.4. Immunohistochemistry

The 3 *μ*m paraffin sections of 4% paraformaldehyde-fixed kidneys were placed in citrate-buffered solution (pH 6.0) and then boiled for 30 min for antigen retrieval. Endogenous peroxidase was blocked with 3% hydrogen peroxide, and nonspecific binding was blocked with 10% BSA. Primary antibodies were as follows: Mouse anti-collagen type III (1 : 100; Abnova, Taipei City, Taiwan), anti-*α*-smooth muscle actin (*α*-SMA) (1 : 100; Thermo Fisher Scientific, Cheshire, UK), anti-CD3 (1 : 50; Thermo Fisher Scientific), anti-ED-1 (1 : 50; AbD Serotec, Oxfordshire, UK), anti-myeloperoxidase (MPO; 1 : 100; Novus Biologicals, Littleton, CO, USA), rabbit anti-Ki-67 (1 : 100; Lab Vision Co., Fremont, CA, USA), and anti-Kv1.3 (1 : 100; Bioss Inc., Woburn, MA, USA). Diaminobenzidine substrate (Sigma Chemical Co., St. Louis, MO, USA) was used for the color reaction. At the end of the staining, the sections were counterstained with hematoxylin. The secondary antibody alone was consistently negative on all sections. Toluidine blue staining was performed by immersion of the sections in 0.1% toluidine blue (Muto Pure Chemical Co., Tokyo, Japan) for 30 min at room temperature. Mast cells were identified by their characteristic metachromasia. For quantitative analysis, the numbers of CD3-, ED-1-, toluidine blue-, MPO-, Ki-67-, and *α*-SMA-positive cells were counted in high-power fields of the cortical interstitium as described in our previous studies [16, 21–23].

### 2.5. Real-Time RT-PCR

Total RNAs from freshly isolated renal cortex were extracted using the RNeasy mini kit (Qiagen, Hilden, Germany). First-stand cDNA was synthesized from 2 *μ*g of total RNA of each tissue in 20 *μ*l of reaction mixture using the SuperScript VILO first-strand synthesis kit (Invitrogen, Carlsbad, CA, USA). The quantitative RT-PCR was carried out using the Applied Biosystems 7500 Real-Time PCR System (Life Technologies Inc, Gaithersburg, MD, USA) with SYBR Premix Ex Taq II (Takara Bio, Kyoto, Japan). The sequences of the primers used were as follows: KCNA3, forward 5′-GCTCTCCCGCCATTCTAAG-3′, reverse 5′-TCGTCTGCCTCAGCAAAGT-3′; GAPDH, forward 5′-GGCACAGTCAAGGCTGAGAATG-3′, reverse 5′-ATGGTGGTGAAGACGCCAGTA-3′. The quantity of RNA samples was normalized by the expression level of GAPDH.

### 2.6. Other Measurements and Statistical Analyses

Serum electrolytes, creatinine, and blood urea nitrogen levels were measured using a chemical autoanalyzer (DRI-CHEM 3500V; Fuji, Tokyo, Japan). Data were analyzed using Microsoft Excel (Microsoft Co., Redmond, WA, USA) and reported as means ± SEM. Statistical significance was assessed by two-way ANOVA followed by Dunnett's or Student's *t*-test. A value of *P* < 0.05 was considered significant.

## 3. Results

### 3.1. Progression of Renal Fibrosis and Leukocyte Proliferation in UUO Rat Kidneys

**Figure 1:**
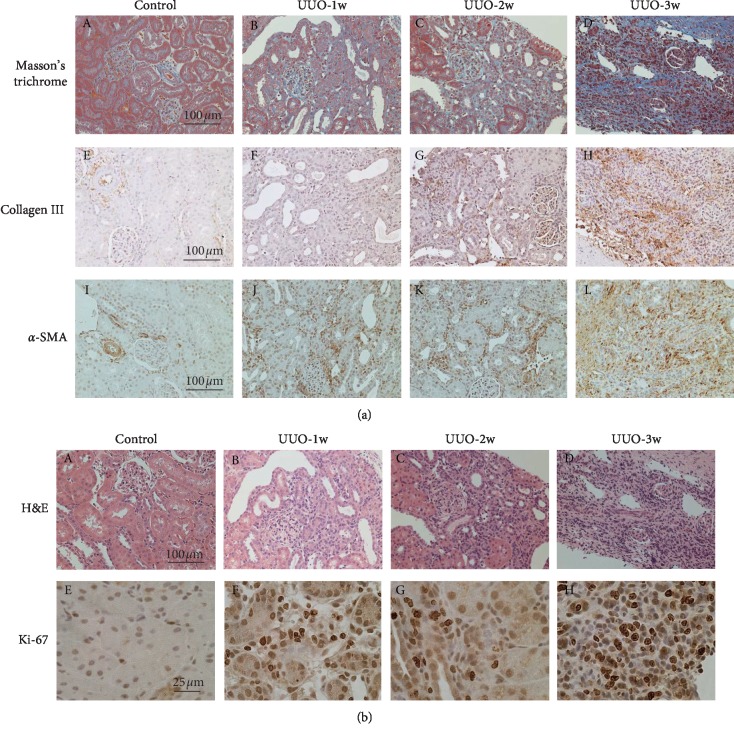
Fibrotic marker expression and leukocyte proliferation in UUO rat kidneys. (a) Masson's trichrome staining and immunohistochemistry using antibodies for collagen III (brown) and *α*-smooth muscle actin (*α*-SMA) (brown) in control (A, E, I) and UUO rat kidneys with 1 week (UUO-1w; B, F, J), 2 weeks (UUO-2w; C, G, K), and 3 weeks (UUO-3w; D, H, L) after unilateral ureteral obstruction. Magnification: ×20. (b) Hematoxylin and eosin (H&E) staining and immunohistochemistry for Ki-67 (brown) in control (A, E) and UUO rat kidneys with 1 week (UUO-1w; B, F), 2 weeks (UUO-2w; C, G), and 3 weeks (UUO-3w; D, H) after unilateral ureteral obstruction. (A–D) Magnification: ×20. (E–H) Magnification: ×60.

Serum creatinine and blood urea nitrogen levels in rats 3 weeks after UUO were compatible with those in normal rats (serum creatinine, 0.43 ± 0.06 mg/dl; blood urea nitrogen, 17.7 ± 0.60 mg/dl; *n* = 4), indicating that renal function was well preserved in UUO rats. However, in these rat kidneys, Masson's trichrome staining and the immunohistochemistry for collagen III, a marker of fibrosis, demonstrated a wide range of staining in the cortical interstitium (Figure 1(a) (A versus B–D), (E versus F–H)), which expanded progressively with the increasing number of postobstructive days. Immunohistochemistry for *α*-SMA, a marker of myofibroblasts, also demonstrated increasing numbers of positively stained cells within the interstitium (Figure 1(a) (I versus J–L)). These results indicated the progression of renal fibrosis in rat kidneys with UUO, which confirmed the propriety of our rat model [9–11]. In the present study, we did not perform urine examination, since the unobstructed contralateral kidneys in UUO models usually offset the loss of renal function [24]. Additionally, previous studies indicated the lack of proteinuria in UUO models because the injured kidneys were completely obstructed and had no urine output [10]. However, by directly collecting the urine from the injured kidneys, recent studies have revealed the presence of several urinary proteins in UUO rat models, which may serve as candidate biomarkers of renal tubular injury and interstitial fibrosis [9].

In UUO rat kidneys, in addition to the progressive tubular dilatation and atrophy (Figure 1(b) (A versus B–D)), there were an increasing number of small round cells in the cortical interstitium, suggesting the increase in the inflammatory leukocytes. Since these small round cells were positive for Ki-67, a marker for cellular proliferation (Figure 1(b) (E–H)), these leukocytes were considered to proliferate *in situ* within the cortical interstitium.

### 3.2. T-Lymphocytes and Macrophages Are Prominently Increased in UUO Rat Kidneys

**Figure 2:**
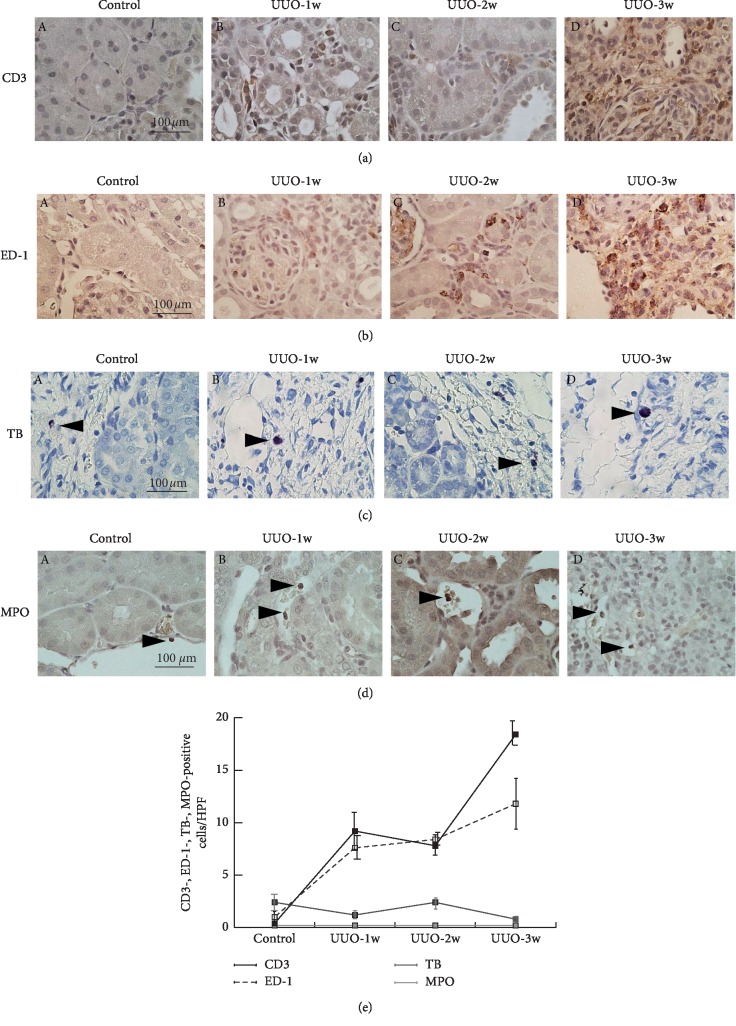
Markers for T-lymphocytes, macrophages, mast cells, and neutrophils expression in the cortical interstitium of UUO rat kidneys. Immunohistochemistry using antibodies for CD3 (a), ED-1 (b) (brown), toluidine blue (TB) staining (c) (blue, arrow heads), and immunohistochemistry for myeloperoxidase (MPO) (d) (brown, arrow heads) in control (A) and UUO rat kidneys with 1 week (UUO-1w, B), 2 weeks (UUO-2w, C), and 3 weeks (UUO-3w, D) after unilateral ureteral obstruction. Magnification: ×60. (e) Numbers of CD3-positive T-lymphocytes, ED-1-positive macrophages, toluidine blue-positive mast cells, and myeloperoxidase-positive neutrophils were counted in high-power views within the cortical interstitium of control, UUO-1w, UUO-2w, and UUO-3w rat kidneys. Values are means ± SEM (*n* = 5).

Previously, in our rat models with advanced CRF, the increased numbers of T-lymphocytes or macrophages in the cortical interstitium primarily contributed to the progression of renal fibrosis, since the cytokines produced by the leukocytes stimulated the fibroblasts' activity to produce collagen [20, 22]. Therefore, we examined the distribution of these leukocytes within the interstitium of the UUO rat kidneys (Figures 2(a) and 2(b)). One or two weeks after the induction of UUO, immunohistochemistry demonstrated the infiltration of some CD3 or ED-1-positive cells within the interstitium (Figures 2(a) (A versus B, C) and 2(b) (A versus B, C)). Then by 3 weeks, most of the interstitial leukocytes were positive for either CD3 or ED-1 (Figures 2(a) (D) and 2(b) (D)), indicating that the increased leukocytes were mainly T-lymphocytes or macrophages. In contrast to these leukocytes, there were only a few toluidine blue-positive mast cells in the renal subcapsular interstitial space (Figure 2(c), arrow heads), which did not increase despite the increasing numbers of postobstructive days (Figure 2(c) (A versus B–D)). Immunohistochemistry for myeloperoxidase demonstrated the presence of only a few positive cells through the observation period (Figure 2(d), arrow heads), indicating few infiltration of neutrophils.  Figure 2(e) depicts the postobstructive changes in the numbers of CD3-, ED-1-, toluidine blue-, and myeloperoxidase-positive cells, which were counted in high-power views of the cortical interstitium. The differences between the numbers of T-lymphocytes or macrophages and those of mast cells or neutrophils became most prominent at 3 weeks after UUO (Figure 2(e)).

### 3.3. Leukocytes Overexpressed Kv1.3-Channels in UUO Rat Kidneys

**Figure 3:**
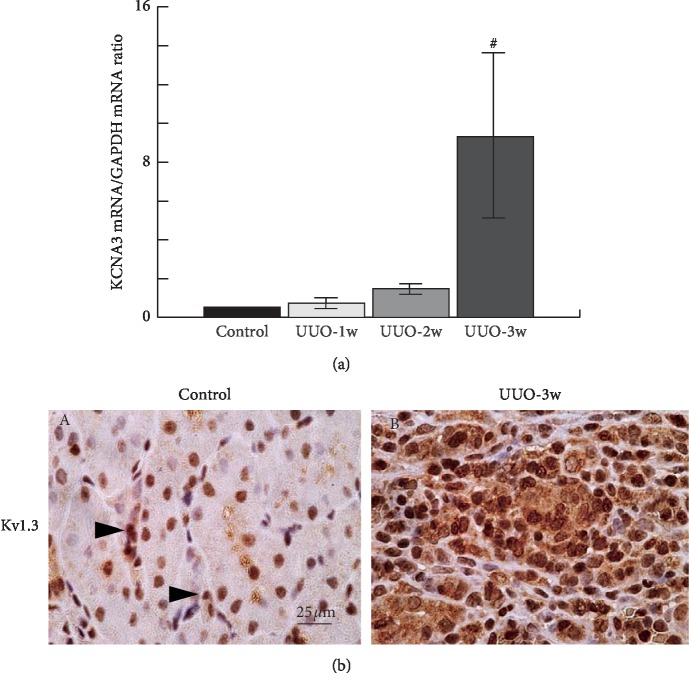
Kv1.3 expression in UUO rat kidneys. (a) KCNA3 mRNA abundance in the renal cortex of control and UUO rat kidneys with 1 week (UUO-1w), 2 weeks (UUO-2w), and 3 weeks (UUO-3w) after unilateral ureteral obstruction. ^#^*p* < 0.05 versus control rats. Values are means ± SEM (*n* = 6). Differences were analyzed by ANOVA followed by Dunnett's or Student's *t*-test. (b) Immunohistochemistry using antibody for Kv1.3 (brown, arrow heads) in control (A) and UUO-3w (B) rat kidneys. High-power views of cortical interstitium. Magnification: ×60.

In addition to megakaryocytes or platelets [25, 26], leukocyte, such as T-lymphocytes, or macrophages, predominantly express Kv1.3-channels in their plasma membranes [27]. These channels play pivotal roles in cellular immunity by facilitating calcium influx required for cellular proliferation and activation [18]. Therefore, we examined the leukocyte expression of Kv1.3-channels in UUO rat kidneys (Figure 3). By 2 weeks after UUO, the expression of Kv1.3 mRNA, KCNA3, was significantly increased in the cortex isolated from the rat kidneys (Figure 3(a)). By 3 weeks after UUO, the expression was dramatically increased, which showed similar patterns to the time-dependent progression of renal fibrosis (Figure 1(a)) and the proliferation of leukocytes (Figures 1(b) and 2). In control rat kidneys, as we previously demonstrated [20], immunohistochemistry for Kv1.3 showed weak staining in the cytoplasm of normal proximal tubular cells (Figure 3(b) A). However, at 3 weeks after UUO, Kv1.3 became overexpressed within the cytoplasm of proliferating leukocytes in the cortical interstitium (Figure 3(b) B).

### 3.4. Therapeutic Effects of a Selective Kv1.3-Channel Inhibitor in UUO Rat Kidneys

In our previous patch-clamp studies, margatoxin, a highly selective Kv1.3-channel inhibitor, almost totally suppressed the channel currents in lymphocytes [28, 29]. In both *in vitro* and *in vivo* studies, this drug was actually demonstrated to repress the proliferation of lymphocytes and their cytokine production [20, 30]. In the present study, to obtain the direct evidence that the overexpression of Kv1.3-channels contributes to the proliferation of leukocytes and to the progression of renal fibrosis, we actually treated the UUO rats with margatoxin and examined the fibrosis or leukocyte marker expression within the kidneys.

#### 3.4.1. Effects of Margatoxin on the Progression of Renal Fibrosis

**Figure 4:**
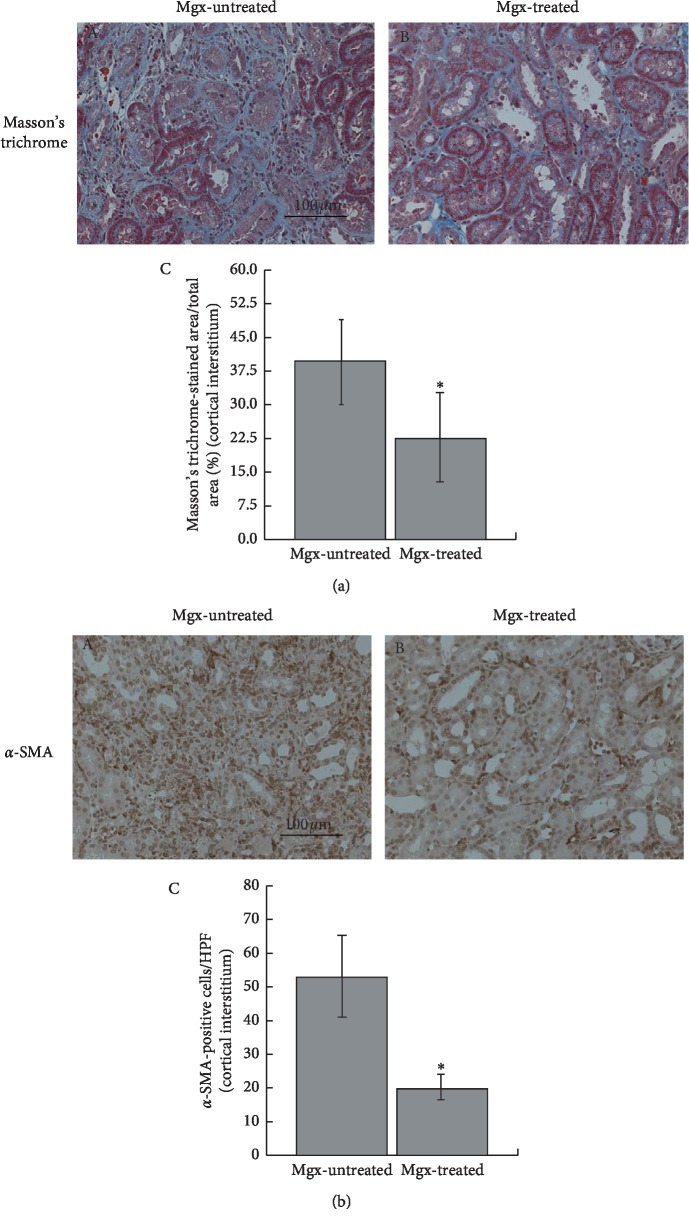
Fibrotic marker expression in margatoxin-untreated and margatoxin-treated UUO rat kidneys. (a) Masson's trichrome staining in margatoxin- (Mgx-) untreated (A) and margatoxin-treated (B) UUO rat kidneys. Low-power views of cortex. Magnification: ×20. (C) Masson's trichrome deposition was quantified and expressed as percentages of Masson's trichrome-positive areas relative to the total areas. (b) Immunohistochemistry using an antibody for *α*-SMA (brown) in margatoxin- (Mgx-) untreated (A) and margatoxin-treated (B) UUO rat kidneys. Magnification: ×20. (C) *α*-SMA-positive cells were counted in high-power views of the cortical interstitium. ^*∗*^*P* < 0.05 versus margatoxin-untreated UUO rats. Values are means ± SEM (*n* = 5). Differences were analyzed by ANOVA followed by Dunnett's or Student's *t* test.

In margatoxin-treated UUO rat kidneys, Masson's trichrome staining demonstrated much smaller size of the cortical interstitium compared to that in margatoxin-untreated kidneys (Figure 4(a) (B versus A)). There was actually a statistical significance in the percentages of the Masson's trichrome-stained areas relative to the total areas between the margatoxin-untreated and margatoxin-treated UUO rat kidneys (Figure 4(a) C). Additionally, immunohistochemistry *α*-SMA demonstrated a significant decrease in the number of myofibroblasts in margatoxin-treated UUO rat kidneys (Figure 4(b) B versus A), which was quantitatively confirmed by the decreased number of *α*-SMA-positive cells in high-power fields (Figure 4(b) C). These results strongly suggested that margatoxin suppressed the number of myofibroblasts and thus halted the progression of renal fibrosis in UUO rat kidneys.

#### 3.4.2. Effects of Margatoxin on Infiltration and Proliferation of Interstitial Leukocytes

**Figure 5:**
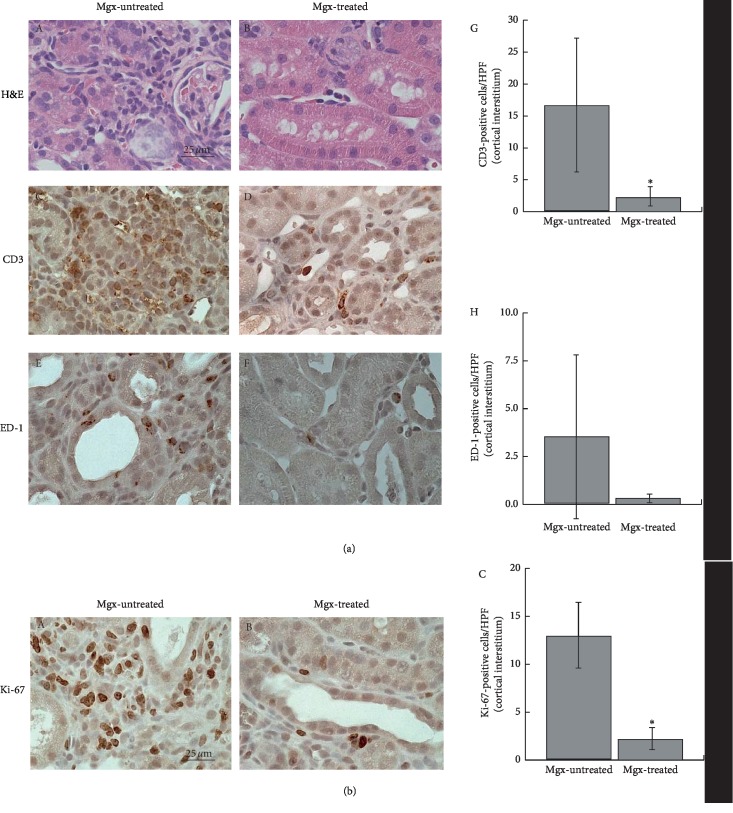
Markers for leukocytes and their proliferation in margatoxin-untreated and margatoxin-treated UUO rat kidneys. (a) Hematoxylin and eosin (H&E) staining and immunohistochemistry using antibodies for CD3 and ED-1 (brown) in margatoxin- (Mgx-) untreated (A, C, E) and margatoxin-treated (B, D, F) UUO rat kidneys. High-power views of cortex. Magnification: ×60. CD3-positive (G) and ED-1-positive (H) cells were counted in high-power views of the cortical interstitium. (b) Immunohistochemistry using antibody for Ki-67 (brown) in margatoxin- (Mgx-) untreated (A) and margatoxin-treated (B) UUO rat kidneys. High-power views of cortical interstitium. Magnification: ×60. (C) Ki-67-positive cells were counted in high-power views of the cortical interstitium. ^*∗*^*P* < 0.05 versus margatoxin-untreated UUO rats. Values are means ± SEM (*n* = 6). Differences were analyzed by ANOVA followed by Dunnett's or Student's *t* test.

In the cortical interstitium of margatoxin-untreated UUO rat kidneys, there were a substantial number of infiltrating leukocytes (Figure 5(a) A), such as CD3-positve T-lymphocytes and ED-1-positive macrophages (Figure 5(a) (C, E)). However, in margatoxin-treated UUO rat kidneys, the numbers of these cells were much smaller in the cortical interstitium (Figure 5(a) (B, D, F)). As shown in Figure 5(a) (G), significant difference was obtained in the number of CD3-positive cells between margatoxin-treated and margatoxin-untreated UUO rat kidneys. These results indicated that margatoxin actually decreased the numbers of inflammatory leukocytes in the renal interstitium of UUO rat kidneys.

Immunohistochemistry for Ki-67 demonstrated a large number of positively stained inflammatory leukocytes within the cortical interstitium of margatoxin-untreated UUO rat kidneys (Figure 5(b) A). However, in margatoxin-treated UUO rat kidneys, there were much less leukocytes positively stained with Ki-67 (Figure 5(b) B). As shown in Figure 5(b) (C), a marked difference was obtained in the numbers of Ki-67-positive cells between margatoxin-treated and margatoxin-untreated UUO rat kidneys. From these results, margatoxin was thought to suppress the *in situ* proliferation of infiltrating leukocytes and thus decreased their numbers within the UUO rat kidneys.

## 4. Discussion

Using animal models of UUO, previous studies revealed the involvement of mast cells in the development renal fibrosis [12–14] because mast cells release growth factors or cytokines that stimulate the collagen synthesis from fibroblasts [15, 16]. In some studies, tranilast, a mast cell stabilizer, actually ameliorated the progression of renal fibrosis in UUO [31, 32]. However, due to their small occupation in leukocytes that infiltrated into the cortical interstitium, targeting mast cells alone was not enough for the treatment. In the pathogenesis of renal fibrosis, transforming growth factor beta-1 (TGF-*β*1) plays a major role, since it directly promotes the fibroblast proliferation and stimulates their collagen synthesis [33]. TGF-*β*1 also activates the downstream Smad signal transduction pathway to generate extracellular matrix [34]. Regarding the mechanism by which tranilast exerted antifibrotic effects [16, 35], this drug was considered to decrease the TGF-*β*1 expression and repress its activity in the fibrotic kidneys [31, 32, 36, 37], in addition to its mast cell-stabilizing properties [16, 38]. In the present study, we clearly demonstrated the predominance of T-lymphocytes or macrophages over mast cells or neutrophils in UUO rat kidneys, which became most prominent at 3 weeks after inducing UUO (Figure 2). In our previous study using rat models with advanced CRF, proinflammatory cytokines produced by the inflammatory leukocytes actually activated fibroblasts to produce collagen [22]. Additionally, in UUO rat kidneys, the time-dependent increase in T-lymphocytes and macrophages was well correlated with such a progression pattern of renal fibrosis (Figures 1(a) and 2). Therefore, these inflammatory leukocytes were thought to be directly responsible for the progression of interstitial renal fibrosis in UUO.

T-lymphocytes and macrophages predominantly express Kv1.3-channels in their plasma membrane [18]. In UUO rat kidneys with longer postobstructive days, these leukocytes overexpressed the Kv1.3-channels within the cortical interstitium of fibrotic kidneys (Figure 3). In previous studies, the overexpression of Kv1.3-channels was noted in isolated cells under certain pathological conditions, such as cancer [39, 40], neuroinflammatory disorder, or ischemic heart disease [41, 42]. In these cells, Kv1.3-channels stimulate calcium signals to facilitate cellular proliferation by generating a driving force for inward calcium flow [17, 43]. In the present study, margatoxin, a selective inhibitor of Kv1.3-channels, suppressed the proliferation of leukocytes (Figure 5) and actually ameliorated the progression of renal fibrosis in UUO rat kidneys (Figure 4). Therefore, as previously shown in cancer cells or neuroinflammatory cells [42, 44], the membrane hyperpolarization bought about by the channels was thought to be responsible for the leukocyte proliferation/activation and the subsequent progression of renal fibrosis. Using a murine model of UUO, Grgic et al. demonstrated a therapeutic usefulness of targeting the intermediate-conductance Ca^2+^-activated K^+^-channels (Kc_a_3.1) in the treatment of renal fibrosis, since these channels were overexpressed in proliferating fibroblasts [45]. From our results, the Kv1.3-channels overexpressed in lymphocytes or macrophages could also be the useful therapeutic target in the treatment of renal fibrosis.

Our recent patch-clamp study revealed that antiallergic drugs, such as cetirizine, fexofenadine, azelastine, and terfenadine, effectively suppressed lymphocyte Kv1.3-channels [46]. These lipophilic drugs were thought to distribute freely into the phospholipid bilayers of cell membrane [46, 47] and thus directly intruded into the composite domains of the channels from inside the membranes. Of note, since azelastine and terfenadine are more lipophilic than the other drugs [47], they would remain within the membranes for a long time, bringing about more continuous inhibitory pattern of the Kv1.3-channels [46]. In previous patch-clamp studies, we also revealed that so-called “commonly used drugs,” such as antimicrobials, anti-hypertensives, and anticholesterol drugs, actually exerted inhibitory properties on the Kv1.3-channel currents in lymphocytes [28, 48–51]. Based on such pharmacological characteristics, we could clinically apply these commonly used drugs in the treatment of renal fibrosis. Since these drugs have long been used in daily medical practice, they are more reliable and safer drugs than the selective channel inhibitors that were chemically synthesized originally from venom or scorpion toxins [52–55].

In summary, this study clearly demonstrated that the numbers of T-lymphocytes and macrophages were markedly increased in UUO rat kidneys at 3 weeks after the operation. The overexpression of Kv1.3-channels in leukocytes was thought to be responsible for the proliferation of these cells and the progression of renal fibrosis. This study strongly suggested the therapeutic usefulness of targeting lymphocyte Kv1.3-channels in the treatment of renal fibrosis.

## Data Availability

The data used to support the findings of this study are available from the corresponding author upon request.
